# Glomangioma of the Renal Artery: A Rare Case of a Glomus Tumor

**DOI:** 10.7759/cureus.91322

**Published:** 2025-08-31

**Authors:** Stavros Grigoriadis, Stavros Spiliopoulos, Konstantinos Palialexis, Athanasios Korogiannos, Zannis Almpanis, Ioannis Paraskevopoulos

**Affiliations:** 1 2nd Department of Radiology, Interventional Radiology Unit, "Attikon” University General Hospital, Athens, GRC; 2 Interventional Radiology-Oncology Clinic, Athens Medical Center, Athens, GRC; 3 2nd Department of Radiology, Interventional Radiology Unit, "Attikon" University General Hospital, Athens, GRC; 4 3rd Department of Medical Oncology, Mediterraneo Hospital, Athens, GRC; 5 Department of Pathology, PathLabs Laboratory, Athens, GRC; 6 Department of Clinical Radiology and Imaging, Faculty of Medicine, University Hospital of Ioannina, Ioannina, GRC

**Keywords:** core biopsy, glomagioma, glomus body, glomus tumor, percutaneous ct-guided biopsy, renal artery

## Abstract

Glomus tumors are rare mesenchymal neoplasms originating from the glomus bodies of the skin. When found in the kidney, these tumors are uncommon, with very few documented cases in the medical literature. Our comprehensive review identified only a very limited number of reported primary renal glomus tumors. Although the majority of these tumors are benign, two cases have been reported as malignant and one as having indeterminate malignant potential.

We describe a rare case: a 69-year-old male patient with a history of transitional cell bladder cancer and possible local recurrence in the anatomical region of the proximal left renal artery, identified as a new finding during scheduled follow-up examinations. The same evaluation also revealed an asymptomatic left hydronephrosis with impaired renal parenchymal function, attributed to previous therapeutic interventions. Histopathological and immunohistochemical analysis confirmed the diagnosis of glomangioma, a type of glomus tumor.

To the best of our knowledge, this represents the 23rd documented case of a primary benign renal glomus tumor, and based on the existing literature, there are no other case reports describing this tumor arising from the renal artery. Given that primary renal glomus tumors are rare and can mimic other mesenchymal kidney neoplasms on imaging, thorough evaluation of any kidney tumor is crucial. This should include detailed histopathological and immunohistochemical studies, as accurate diagnosis is vital for patient management, since these tumors typically follow a benign course after surgical removal.

## Introduction

An unusual benign neoplasm arising from the neuro-arterial plexus is known as a glomus tumor. The glomus body is a specialized arteriovenous anastomosis that operates without an intervening capillary network, functioning primarily in temperature regulation. Although glomus tumors are infrequent, representing under 2% of all soft tissue tumors [1], they are most often seen in women between 20 and 40 years of age [1-5]. They typically present with the classic triad of paroxysmal pain, cold sensitivity, and pinpoint tenderness, particularly when located in the subungual region.

Extra-digital glomus tumors are exceptionally rare, as glomus bodies are typically absent from visceral organs [1-4]. Consequently, the occurrence of a glomus tumor in an organ such as the kidney presents a significant diagnostic challenge. These tumors can easily be mistaken for more common renal neoplasms, leading to delays in diagnosis and treatment [5]. To date, only 22 cases of primary renal glomus tumors have been documented in the literature following an extensive review [1, 6]. Furthermore, the current 4th edition of the WHO classification system for kidney tumors omits pericytic tumors and the extremely rare glomus tumors, which can contribute to diagnostic difficulty.

The present case report describes the 23rd reported case of a glomus tumor of the kidney [1-6]. Based on current knowledge, it is the first documented case to arise from the renal artery. This finding is significant because it provides new insights into the pathogenesis and clinical behavior of these rare tumors. By detailing the diagnostic process and clinical course of this unusual presentation, we hope to improve the understanding of glomus tumors in atypical locations. Recognition of these tumors is critical, as confusion with malignant tumors can lead to unnecessary and potentially dangerous surgical procedures or invasive interventions that carry a risk of hemorrhage. This case underscores the importance of considering rare differential diagnoses and highlights a potential need for revisions in current classification systems to include these uncommon tumors [7-9], ultimately leading to more timely and accurate diagnoses for patients.

## Case presentation

**Figure 1:**
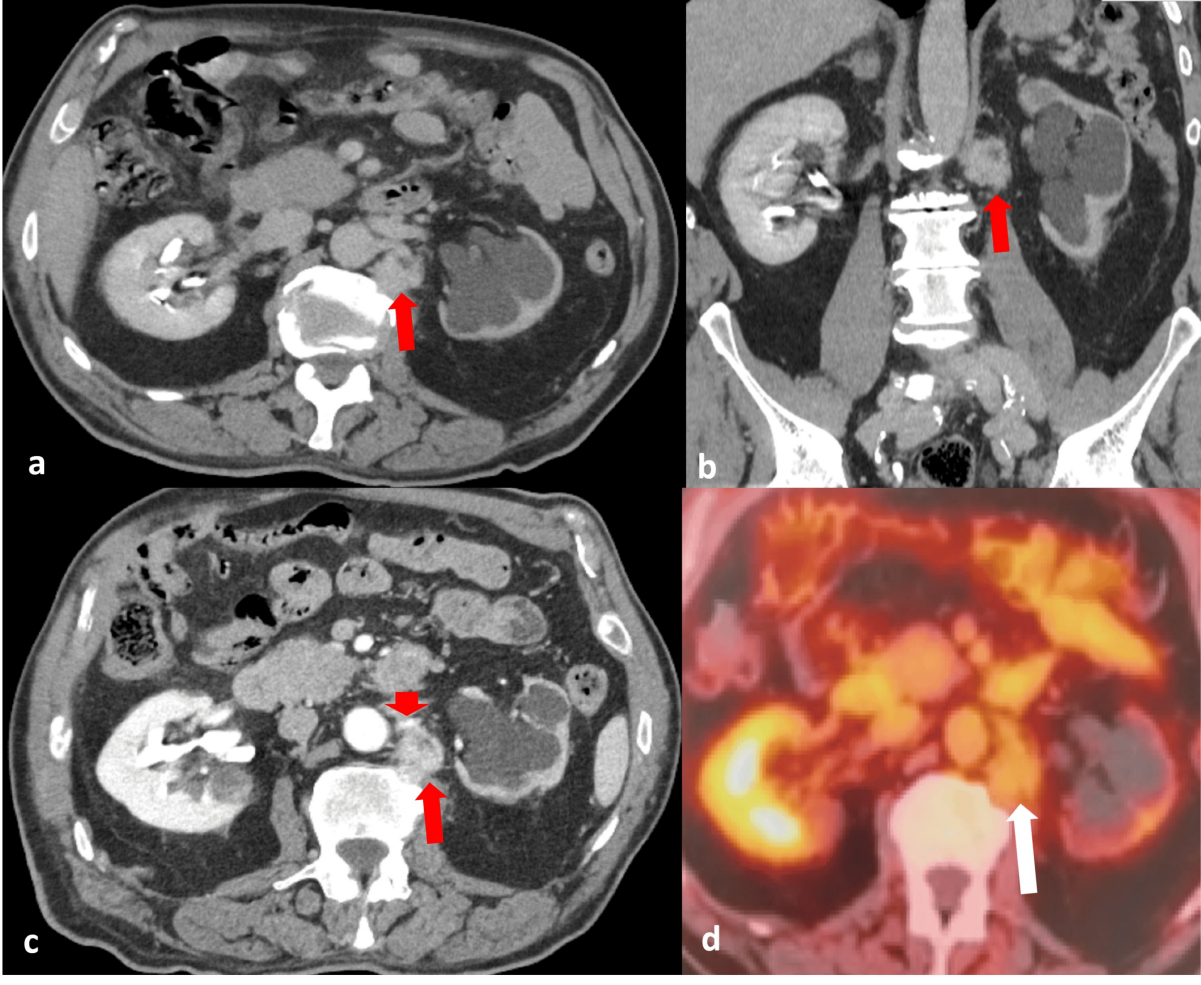
(a) Relatively well-defined lesion at the proximal left renal artery (arrow) in axial view (portal-venous phase) and (b) in coronal view (portal-venous phase). (c) Arterial phase showing the same relatively well-defined, heterogeneously enhancing lesion at the proximal left renal artery (arrow) in axial view. Note the lesion’s close proximity to the left renal artery (arrowhead). (d) PET-CT image of the same lesion at the left renal artery (arrow), measuring 2.2 × 1.8 cm with an SUVmax of 2.8. SUVmax: Maximum Standardized Uptake Value.

**Figure 2:**
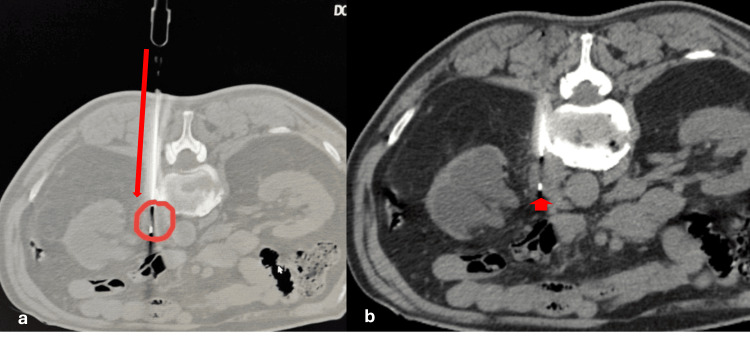
(a-b) Axial images of the access route during CT-guided coaxial core-needle biopsy of the left para-aortic mass. Note the needle’s access path (arrow) and the tip of the core needle within the lesion (circle in 2a and arrowhead in 2b), verified just before sampling.

An unusual benign neoplasm arising from the neuro-arterial plexus. A 69-year-old male patient, who had previously undergone surgery and chemotherapy for transitional cell urinary cancer, presented for a scheduled 18-month follow-up. Abdominal CT and PET-CT scans (Figures 1a-1d) demonstrated a relatively well-defined, heterogeneously enhancing lesion at the proximal left renal artery, measuring 2.2 × 1.8 cm with an SUVmax of 2.8. A percutaneous biopsy was recommended during the oncologic multidisciplinary team (MDT) meeting. Under CT guidance, a coaxial fine core-needle biopsy of the left para-aortic mass was performed, utilizing an 18-cm long, 16G needle (Figures 2a-2b).

**Figure 3:**
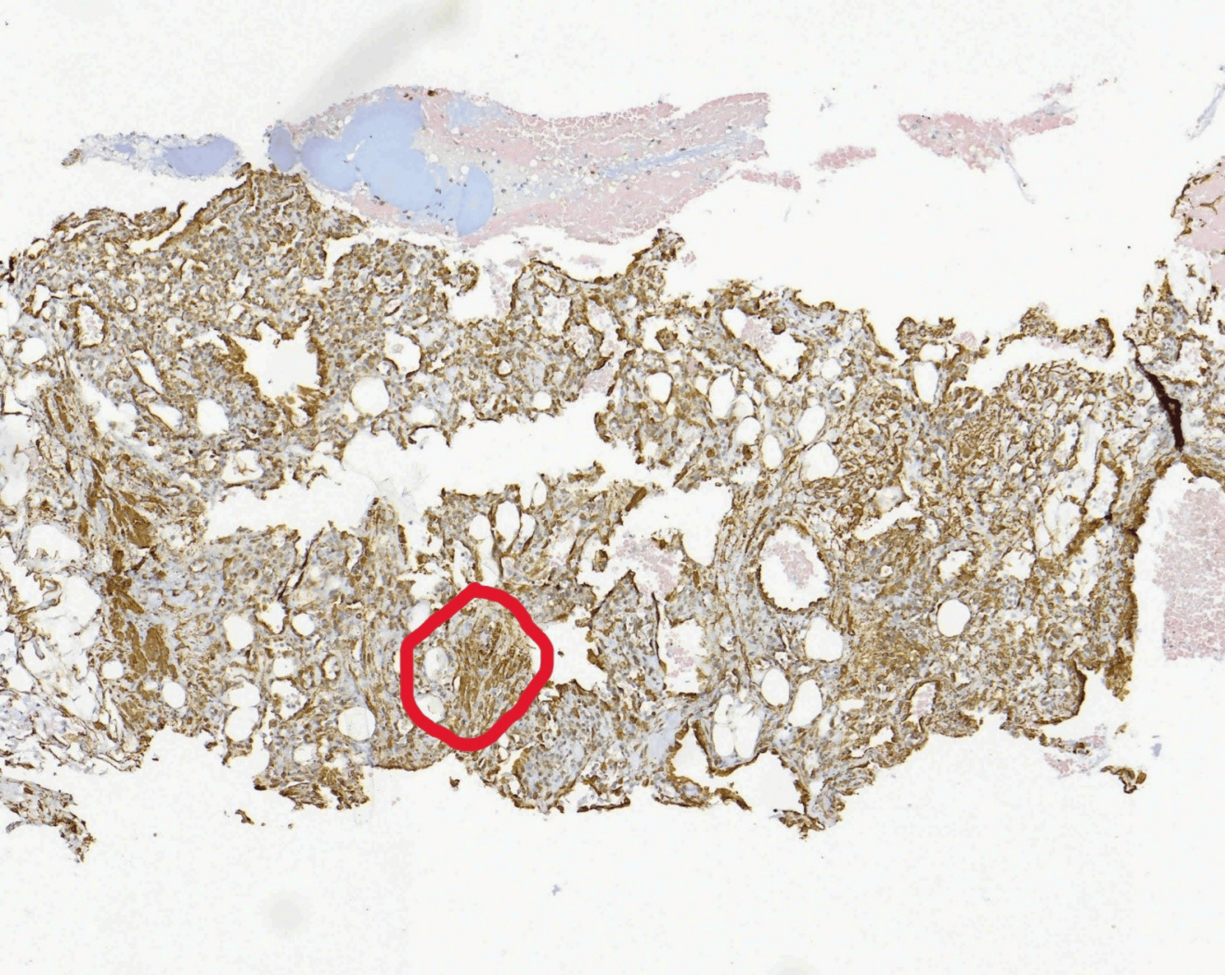
Glomus cells, small and uniform with well-defined cell borders (red circle).

**Figure 4:**
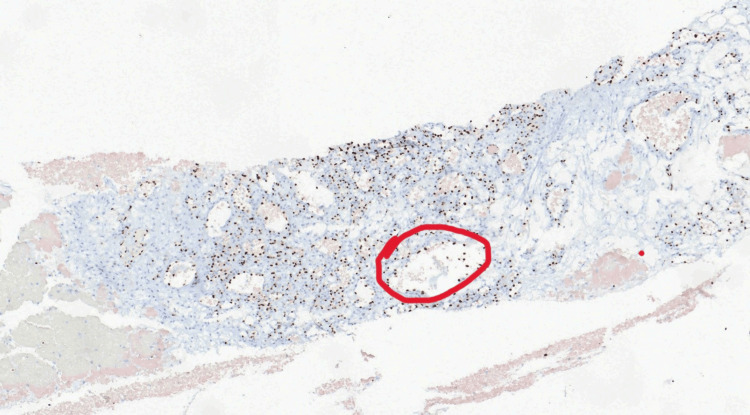
Thin-walled, capillary-sized vessels without atypia (red circle).

**Figure 5:**
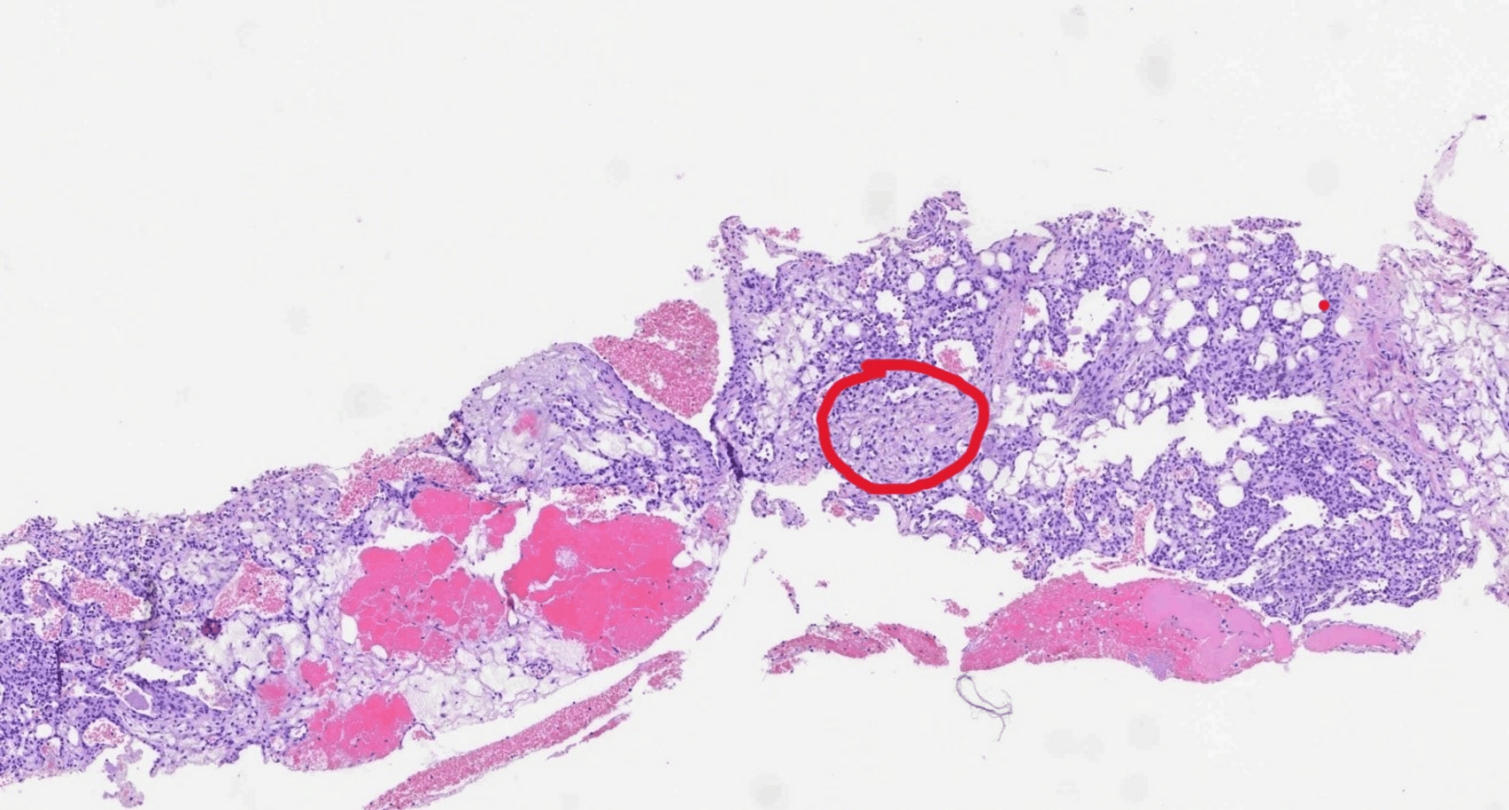
Smooth, elongated muscle cells without atypia (red circle).

In total, two filamentous tissue fragments of brownish-hemorrhagic color and fibroelastic consistency, measuring 1.7 cm and 1.5 cm in length, were obtained and sent for histological examination. The examined specimen was reported to be a generally benign pericytic (perivascular) kidney lesion, consisting histologically of three components: glomus cells, which were small and uniform with well-defined cell borders (H&E stain, Figure 3); blood vessels, which were thin-walled, capillary-sized, and without atypia (positive ERG stain, Figure 4); and smooth muscle cells, which were elongated and without atypia (positive SMA stain, Figure 5).

The above histomorphological findings were suggestive of glomangioma/glomus tumor of the kidney.

The decision not to proceed with surgical resection was made after an MDT meeting that included a medical oncologist, a surgical oncologist, and a radiologist. The primary therapeutic focus remained on managing the patient’s malignant transitional cell carcinoma rather than the benign glomangioma. Given the patient’s poor overall clinical status at the time, the MDT reached a consensus to prioritize palliative chemotherapy. Surgical intervention remained a potential option, contingent on significant improvement in the patient’s general condition and a favorable response to chemotherapy. Unfortunately, the patient’s clinical decline precluded further consideration of resection. He continued chemotherapy and passed away approximately six months later due to complications of the malignant disease.

## Discussion

Glomus bodies, which are specialized arteriovenous physiological structures, have a dense nerve supply. The first description of a glomus tumor was made by Masson in 1924. These tumors are perivascular mesenchymal neoplasms composed of cells that closely resemble the modified smooth muscle cells of normal glomus bodies. Glomus tumors are found in both sexes with nearly equal frequency, though they are slightly more common in females, particularly in subungual locations. They are most often seen in young adults between 20 and 40 years old. Typically, they present as small, reddish-blue nodules on the extremities, especially under the nails, and are accompanied by pain [5, 7, 8, 9]. Surgical excision and ablation methods are the standard approaches, and the prognosis for typical glomus tumors is excellent [2, 6, 8].

It is notable that approximately 25% of these tumors develop in internal organs, which typically do not contain glomus bodies. This can make diagnosis challenging. The exact process by which glomus tumors form in these internal organs is not well understood, as most originate from soft tissues with normal glomus structures. A small number of primary glomus tumors have been reported in the medical literature in locations such as the female reproductive system, digestive system, bones, lungs, mediastinum, larynx, trachea, oral cavity, pancreas, liver, and sinonasal area [1, 2, 7]. Glomus tumors of the kidney are rare, with very few cases reported. Most of these renal tumors are benign. A total of 23 cases have been documented, including the one presented here, with uncertain malignant potential in one case and malignant transformation in two cases [1, 2, 6]. The majority of benign renal glomangiomas have been found in adults between 32 and 81 years of age, with a male-to-female ratio of 2:1. In many cases, patients with glomus tumors show no specific symptoms, or the tumors are detected incidentally during check-ups. Clinical signs, when present, may include mild abdominal pain, flank pain, urinary symptoms, and microscopic hematuria.

Typically, glomus tumors are found in the renal parenchyma, the functional tissue of the kidney. However, there have been rare instances of glomus tumors arising in unusual locations [1-6]. A notable case was reported where a glomus tumor was incidentally discovered in a congenitally hypoplastic kidney. Although 99% of glomus tumors are solitary and occur mainly in adults, around 10% are multiple, as seen in familial generalized multiple glomangiomatosis. This condition primarily affects children, is inherited in an autosomal dominant manner, and shows incomplete penetrance [7-9].

The range of possible differential diagnoses includes solitary fibrous tumor, myopericytoma, paraganglioma, angiomyolipoma, renal hemangioma, juxtaglomerular cell tumor, carcinoid tumor, and lymphoma. Other considerations include Ewing sarcoma, leiomyoma, and renal cell carcinoma [7, 9].

Percutaneous image-guided biopsy is the only indicated procedure for the diagnosis of such lesions situated deep within the retroperitoneal space. Notably, CT-guided fine-needle biopsy (FNB) was technically demanding and carried a high risk of bleeding due to the lesion’s proximity to the renal artery (Figure 1c). However, the biopsy was performed at a specialized vascular center prepared to provide life-saving percutaneous transcatheter embolization in case of hemorrhage.

Based on existing literature, this represents the first histologically proven case of a primary renal glomus tumor arising from the renal artery. Radiologically, these tumors can mimic other mesenchymal neoplasms of the kidney, posing a diagnostic challenge. This challenge is further compounded by the histological and immunohistochemical similarities between glomus tumors and other renal tumors, which can lead to misdiagnosis. Therefore, a thorough investigation, including advanced imaging, image-guided biopsy, and detailed histopathological and immunohistochemical analysis of any incidentally found renal tumors, is crucial for accurate identification, as glomus tumors typically follow a benign clinical course after resection.

## Conclusions

The occurrence of primary renal glomus tumors, of which we present the 23rd case (renal glomangioma), is infrequent and, to our knowledge, this is the first reported case arising from the renal artery. Radiologically, these tumors can mimic other mesenchymal neoplasms of the kidney, posing a diagnostic challenge. This challenge is further compounded by the histological and immunohistochemical similarities between glomus tumors and other renal tumors, which can lead to misdiagnosis. Consequently, a thorough evaluation of any incidentally detected kidney tumor is essential for accurate identification of a glomus tumor. This evaluation should include advanced imaging and, most importantly, image-guided biopsy followed by detailed histopathological and immunohistochemical studies. Such an approach is crucial, as glomus tumors usually follow a benign course after surgical removal. Our case highlights the critical importance of interventional radiology in securing a definitive diagnosis for these rare and complex renal masses.

## References

[1] Almaghrabi A, Almaghrabi N, Al-Maghrabi H (2017). Glomangioma of the kidney: a rare case of glomus tumor and review of the literature. Case Rep Pathol.

[2] Kapogiannis F, Tsiampa E (2021). Glomus tumor of the kidney harboring malignant potential. Cureus.

[3] Sasaki K, Bastacky SI, Hrebinko RL, Parwani AV, Zynger DL (2011). Glomus tumor of the kidney: case report and literature review. Int J Surg Pathol.

[4] Dee E, Loghin A, Toth T, Năznean A, Borda A (2018). Glomus tumor of the kidney: case report. Acta Marisiensis - Seria Medica.

[5] Lazor JW, Guzzo TJ, Bing Z, Lal P, Ramchandani P, Torigian DA (2016). Glomus tumor of the kidney: a case report with CT, MRI, and histopathological findings. Open J Urol.

[6] Gombos Z, Zhang PJ (2008). Glomus tumor. Arch Pathol Lab Med.

[7] Bolognia JL, Jorizzo JL, Rapini RP (2009). Dermatology.

[8] El-Naggar AK, Chan JC, Rubin G, Takata T, Slootweg PJ (2017). WHO Classification of head and neck tumours. https://catalog.nlm.nih.gov/discovery/fulldisplay?docid=alma9917043983406676&context=L&vid=01NLM_INST:01NLM_INST&lang=en&adaptor=Local%20Search%20Engine&tab=LibraryCatalog&query=creator,equals,International%20Agency%20for%20Research%20on%20Cancer,AND&mode=advanced&offset=110.

[9] Walter B (2004). Fitzpatrick's Dermatology in General Medicine.

